# Human Toll-like Receptor 8 (TLR8) Is an Important Sensor of Pyogenic Bacteria, and Is Attenuated by Cell Surface TLR Signaling

**DOI:** 10.3389/fimmu.2019.01209

**Published:** 2019-05-31

**Authors:** Siv H. Moen, Birgitta Ehrnström, June F. Kojen, Mariia Yurchenko, Kai S. Beckwith, Jan E. Afset, Jan K. Damås, Zhenyi Hu, Hang Yin, Terje Espevik, Jørgen Stenvik

**Affiliations:** ^1^Centre of Molecular Inflammation Research, Norwegian University of Science and Technology, Trondheim, Norway; ^2^Department of Clinical and Molecular Medicine, Norwegian University of Science and Technology, Trondheim, Norway; ^3^Department of Infectious Diseases, Clinic of Medicine, St. Olavs Hospital HF, Trondheim University Hospital, Trondheim, Norway; ^4^Clinic of Laboratory Medicine, St. Olavs Hospital HF, Trondheim University Hospital, Trondheim, Norway; ^5^Department of Chemistry and Biochemistry and BioFrontiers Institute, University of Colorado Boulder, Boulder, CO, United States; ^6^School of Pharmaceutical Sciences, Tsinghua University-Peking University Joint Center for Life Sciences, Beijing Advanced Innovation Center for Structural Biology, Tsinghua University, Beijing, China

**Keywords:** human TLR8, bacteria, monocytes, cytokines, IRAK-1

## Abstract

TLR8 is an endosomal sensor of RNA degradation products in human phagocytes, and is involved in the recognition of viral and bacterial pathogens. We previously showed that in human primary monocytes and monocyte derived macrophages, TLR8 senses entire *Staphylococcus aureus* and *Streptococcus agalactiae* (group B *streptococcus*, GBS), resulting in the activation of IRF5 and production of IFNβ, IL-12p70, and TNF. However, the quantitative and qualitative impact of TLR8 for the sensing of bacteria have remained unclear because selective inhibitors have been unavailable. Moreover, while we have shown that TLR2 activation attenuates TLR8-IRF5 signaling, the molecular mechanism of this crosstalk is unknown. We here used a recently developed chemical antagonist of TLR8 to determine its role in human primary monocytes challenged with *S. aureus*, GBS, *Streptococcus pneumonia, Pseudomonas aeruginosa*, and *E. coli*. The inhibitor completely blocked cytokine production in monocytes stimulated with TLR8-agonists, but not TLR2-, and TLR4-agonists. Upon challenge with *S. aureus*, GBS, and *S. pneumonia*, the TLR8 inhibitor almost eliminated the production of IL-1β and IL-12p70, and it strongly reduced the release of IL-6, TNF, and IL-10. With *P. aeruginosa* infection, the TLR8 inhibitor impaired the production of IL-12p70 and IL-1β, while with *E. coli* infection the inhibitor had less effect that varied depending on the strain and conditions. Signaling via TLR2, TLR4, or TLR5, but not TLR8, rapidly eliminated IRAK-1 detection by immunoblotting due to IRAK-1 modifications during activation. Silencing of IRAK-1 reduced the induction of IFNβ and TNF by TLR8 activation, suggesting that IRAK-1 is required for TLR8-IRF5 signaling. The TLR-induced modifications of IRAK-1 also correlated closely with attenuation of TLR8-IRF5 activation, suggesting that sequestration and/or modification of Myddosome components by cell surface TLRs limit the function of TLR8. Accordingly, inhibition of CD14- and TLR4-activation during *E. coli* challenge increased the activation of IRF5 and the production of IL-1β and IL-12p70. We conclude that TLR8 is a dominating sensor of several species of pyogenic bacteria in human monocytes, while some bacteria attenuate TLR8-signaling via cell surface TLR- activation. Taken together, TLR8 appears as a more important sensor in the antibacterial defense system than previously known.

## Introduction

Toll-like receptors (TLR) sense distinct pathogen associated molecular patterns (PAMPs) and initiate inflammatory reactions important for innate and adaptive defense. Humans with genetic defects in the central TLR/IL-1R signaling adaptors MyD88- or IRAK-4 have increased susceptibility to pyogenic bacterial infections, but only during infancy and early childhood (1, 2). On the other hand, excessive inflammation via uncontrolled TLR signaling can initiate sepsis, a syndrome defined as a dysregulated host response resulting in life-threatening organ failure (3). Inhibition of pro-inflammatory sensors and mediators of the host is protective in several animal models of sepsis, yet multiple clinical trials have failed (4). Therefore, there is a need to improve our understanding of the cell host-pathogen interactions, and to clarify which host responses that are protective and which that have adverse effects. This can aid in the identification of new targets and strategies for prevention or treatment of sepsis.

Human TLR8 is highly expressed as a functional cleavage product in endosomes of monocytes and macrophages (5). Mechanistically, the RNA degradation products uridine and short oligomers bind cooperatively at two distinct sites in the N-terminal domain. This induce a conformational change of the pre-formed TLR8-dimer leading to MyD88 recruitment and signaling. Small-molecule agonists such as CL075 have high affinity to the uridine binding site, and is capable in activating TLR8 without RNA oligomers (6). Rodent TLR8 differs structurally and is not activated by these ligands (7), but can be activated in neurons by endogenous microRNA which regulate neuropathic pain (8).

The impact of TLR8 during infection is unclear because neither small animal models nor selective and efficient inhibitors have been available. We previously showed that TLR8 senses entire *S. aureus* and GBS in primary monocytes and macrophages, resulting in the activation of IRF5 and production of IFNβ, IL-12p70, and TNF (9, 10). RNA is likely the bacterial structure required for TLR8 activation, as enzymatic degradation of RNA in *S. aureus* lysates (9) or in GBS upon heat-inactivation strongly attenuate cytokine induction (10). Bacterial RNA is also considered a vita PAMP, a marker of microbial viability (11). Others have shown that TLR8 also contributes in IL-6 production during infection with *Streptococcus pyogenes* (group A *streptococcus*, GAS) (12) and *Escherichia coli* (11, 13) in human myeloid cells. A weakness of these studies is the reliance on molecular tools with limited efficacy and specificity (e.g., siRNA and non-selective inhibitors), and model systems using cell lines do not accurately reflect the role of TLR8 in human primary cells. Thus, the quantitative and qualitative role of TLR8 for the sensing of bacteria needs further clarification. We also revealed that activation of TLR2 negatively regulates TLR8-IRF5 signaling (9). Consequently, bacteria that express high levels of TLR2-agonistic lipoproteins can avoid detection via TLR8, but the molecular mechanism behind this negative TLR-TLR crosstalk is still unknown.

A chemical antagonist of human TLR8 (CU-CPT9a) with high selectivity and efficiency was recently developed (14). CU-CPT9a binds close to the uridine/CL075 binding site in the N-terminal domain and locks TLR8 in the resting state. We here used CU-CPT9a to clarify the role of human TLR8 during bacterial challenge of primary monocytes. Our data show that TLR8 is the dominating sensor of Gram-positive pyogenic bacteria that are major human pathogens. TLR8 also participates in the sensing of the pyogenic Gram-negative species *P. aeruginosa* and *E. coli*. We further show that TLR8 signaling requires IRAK-1 expression, and that cell surface TLR activation attenuates TLR8 signaling, likely via a mechanism involving IRAK-1 and/or other Myddosome components.

## Materials and Methods

### Materials

The TLR8 antagonist CU-CPT9a is previously described (14) and was provided by The Regents of the University of Colorado, a body corporate for and on behalf of the University of Colorado Boulder. The TLR-agonists FSL-1 (TLR2/6), CL075 and polyuridylic acid (polyU; TLR8), ultrapure LPS O111:B4 (TLR4), and purified flagellin from *P. aeruginosa* (TLR5) were purchased from Invivogen. Poly-L-arginine (pLA), the IRAK-4 inhibitor (PF-06426779), and the proteasome inhibitor MG132 were from Sigma-Aldrich (Merck). Humanized anti-CD14 and IgG2/4 control were generously provided by prof. Tom Eirik Mollnes (University of Oslo, Oslo, Norway). BioPlex cytokine assays were from Bio-Rad, and the cytokine levels were determined as per the manufacturer's instructions using Bio-Plex Pro™ Reagent Kit III and the Bio-Plex™ 200 System.

### Bacteria

The bacterial strains GBS NEM316, *S. aureus* 113/113Δ*lgt*, and *S. aureus* Cowan were generously provided by professors Philipp Henneke (University of Freiburg, Germany), Friedrich Göetz (University of Tübingen, Tübingen, Germany), and Timothy Foster (Trinity College, Dublin, Ireland), respectively. The *E. coli* Seattle 1946 strain was obtained from the American Type Culture Collection (ATCC 25922), while *E. coli* and ClearColi® BL21 (DE3) strains were from Lucigen Corporation (USA). Anonymized clinical isolates of GBS, *S. aureus, S. pneumoniae, P. aeruginosa*, and *E. coli* were from a diagnostic collection by the Department of Medical Microbiology, St. Olavs Hospital, Trondheim, Norway. The bacteria were grown on Tryptic soy agar (TSA) or blood agar. For challenge experiments, colonies of *E. coli, S. aureus* and *P. aeruginosa* were grown in Tryptic Soy Broth, while GBS were grown in Todd-Hewitt Broth during vigorously shaking at 37°C. *S. pneumoniae* were grown in Brain-Heart Infusion broth at 37°C and 5% CO_2_. Overnight cultures were diluted 1:100 in fresh broth and grown to exponential phase (~4 h). Bacteria were quantified by optical density, as previously described (10), and the MOI was calculated according to the corresponding CFU counts.

### Monocyte Isolation and Challenge

Human buffycoats and serum were from the Blood bank at St. Olavs Hospital (Trondheim, Norway), with approval by the Regional Committee for Medical and Health Research Ethics (REC Central, Norway, no. 2009/2245). PBMC were isolated using Lymphoprep as described by the manufacturer (Axis Shield Diagnostics, Scotland). Monocytes were purified by adherence in culture plates and maintained in RPMI 1640 (Life Technologies) supplemented with 10% pooled human serum. The cells were pre-incubated with the TLR8 antagonist CU-CPT9a and the control reagent for 2 h, and with the other inhibitors for 30 min. Subsequently, the cells were challenged with bacteria or TLR-agonists, and Gentamicin (100 μg/ml) was added 1 h after the initiation of the challenge to kill extracellular bacteria. Supernatants were stored at −20°C until analyses. THP-1 cells overexpressing recombinant TLR8 was used as previously described (10).

### Immunofluorescence and scanR Analysis

Immunofluorescence labeling and analyses with scanR high-content screening system (Olympus) was done as previously described (9, 10). Primary antibodies used were mouse anti-human IRF5 mAb (Abcam, 10T1, ab33478), rabbit anti-human p65/RelA XP-mAb Cell Signaling Technologies (CST # 8242), and rabbit anti-human p65A (Santa Cruz Biotechnology, #sc-109).

### Western Blotting

Cells were collected and lysed in buffer [1% IGEPAL CA-630, 150 mM NaCl, 50 mM Tris-HCl, pH 7,5, 10% glycerol, 1 mM NaF, 2 mM Na_3_VO_4_, and a protease-phosphatase inhibitor (Complete Mini tablets, Basel, Switzerland)]. Cell lysates were mixed with NuPage LDS sample buffer (Invitrogen) supplemented with 25 mM DTT and denatured at 70°C for 10 min. The samples were separated on 10% Bis-Tris polyacrylamide gels and transferred to nitrocellulose membranes using the iBlot Dry Blotting System (Invitrogen). The membranes were blocked with 5% bovine serum albumin diluted in Tris-buffered saline containing 0.05% Tween-20. Antibodies used were anti-IRAK-1 (CST D51G7, #4504), anti-IRAK-2 (CST #4367) and anti-IRAK-4 (CST #4363), anti-P-Ser396-IRF3 (CST, 4D4G, #4947), and anti-IkBα (CST, 44D4, #4812). Monoclonal anti-GAPDH (Abcam #8245) or anti-beta-tubulin (Abcam, ab6046) were used as loading controls. After incubation with horseradish peroxidase-conjugated secondary antibodies (DAKO), the images were developed using Super Signal West Femto Maximum Sensitivity Substrate (Thermo Scientific) and Odyssey FC Imaging System (LI-COR). Quantification was done using the Image Studio software.

### Gene Silencing and Quantitative PCR

Monocytes were differentiated to macrophages (MDMs) for 5 to 6 days in RPMI 1,640 with 30% pooled human serum. Medium was replaced with RPMI 1640 containing 10% serum before siRNA treatment. A pool of four individual ON-TARGETplus siRNAs (Dharmacon) was transfected using siLentFect (Bio-Rad), yielding a final concentration of 5 nM siRNA. The transfection was repeated after 3 days, and the silenced MDMs were challenged with TLR8 ligand. RNA was isolated with RNeasy including DNAse treatment (Qiagen), cDNA was transcribed with the Maxima cDNA synthesis kit (Thermo Fisher Scientific), and quantitative PCR was done with StepOnePlus using TaqMan probes (Life Technologies) and Perfecta qPCR FastMix (Quanta). The probes used were Hs01077958_s1 (IFNβ), Hs00174128_m1 (TNF), Hs01018347_m1 (IRAK-1). TBP (Hs00427620_m1) served as endogenous mRNA control, and relative expression was calculated using the ΔΔCt method, and plotted as fold induction by stimulation.

### Statistics

Data from independent experiments with monocytes from different donors was used for the statistical calculations, indicated as N (number of experiments). Data was log-transformed to increase the likelihood of a Gaussian data distribution, as this is required for parametrical tests. Data sets with one factor were analyzed by one-way repeated-measures (RM) analysis of variance (ANOVA) and Dunette's multiple comparison test, while data sets with two factors were analyzed by two-way RM ANOVA and Bonferroni's multiple comparison test. For data sets with missing values a mixed model analysis was used. For some samples the cytokine levels were below the limit of detectionand these conditions were excluded from the analysis as indicated with the symbol “v.” Significance levels are indicated as: ^*^p < 0.05, ^**^p < 0.01, and ^***^p < 0.001. Graphs and analyses were generated with GraphPad Prism (v8.01).

## Results

### The Role of TLR8 in the Sensing of *S. aureus*, GBS, and *E. coli* by Human Primary Monocytes

**Figure 1:**
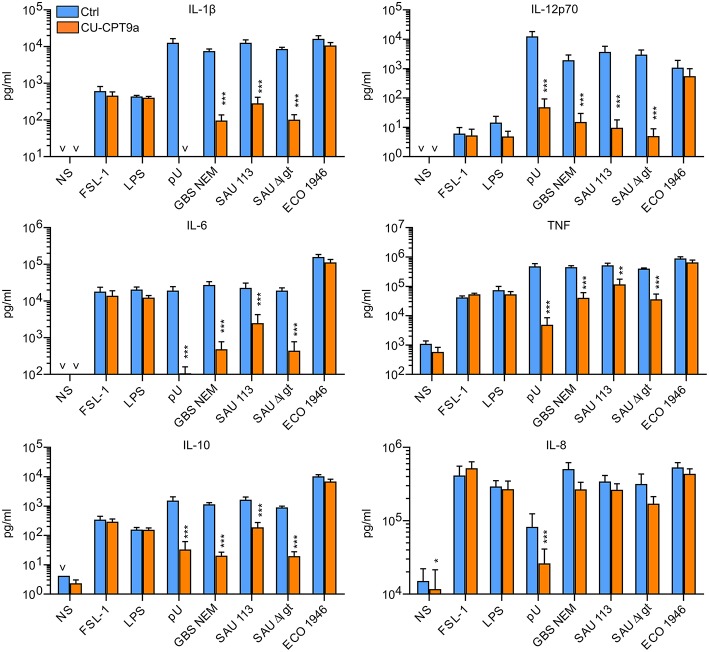
TLR8 inhibition strongly attenuates cytokine production from human primary monocytes challenged with Group B streptococcus (GBS) and *S. aureus*, but not a strain of *E. coli*. Monocytes were pre-treated with control reagent (Ctrl, 5 μM) or TLR8 antagonist (CU-CPT9a, 5 μM) and challenged with TLR8-ligand pU (polyU/poly-L-arginine, 1 μg/ml), TLR2-ligand FSL-1 (100 ng/ml), TLR4-ligand LPS (O111:B4, 100 ng/ml), or live GBS (NEM316), *S. aureus* wild-type (SAU 113) and *lgt*-deficient (SAU 113Δ*lgt*), or *E. coli* Seattle 1946 (ECO1946) for 22 h. Bacterial doses were 1 × 10^6^/ml for GBS and *S. aureus* (MOI 0.1 for GBS, 0.2 for SAU), and 2 × 10^5^/ml for *E. coli* (MOI 0.2). Graphs show mean + SEM (*N* = 4). NS, no stimuli. *p < 0.05, **p < 0.01, and ***p < 0.001.

We here examined the role of TLR8 in the sensing of live bacteria by human primary monocytes using CU-CPT9a, a recently developed small-molecule inhibitor of TLR8 which does not affect the activation of other human TLRs (14). To determine the optimal dose of the inhibitor, we pre-treated monocytes with serial dilutions of CU-CPT9a and challenged the cells with the TLR8 ligands pU/pLA (polyuridylicacid/poly-L-arginine) and CL075. CU-CPT9a completely blocked the cytokine production at 2.5–5 μM while the control compound (Ctrl) had no effect (Figure S1). Cell viability analysis of monocytes revealed that 5 μM CU-CPT9a did not induce cell death (data not shown). To determine the impact of TLR8 in the sensing of live bacteria, we blocked TLR8 activation using 5 μM CU-CPT9a and challenged monocytes with GBS (GBS NEM316) and *S. aureus* 113 (SAU 113) at two doses for 6 and 22 h. We also included an isogenic *lgt*-deficient strain of *S. aureus* which lacks TLR2-stimulatory lipoproteins (SAU 113Δ*lgt*) (15), and an *E. coli* reference strain (Seattle 1946, ECO 1946). TLR2-, TLR4-, and TLR8-ligands were included as controls of specificity and efficiency. CU-CPT9a inhibited the cytokine induction by pU/pLA stimulation completely, but had no effect on cytokine induction by LPS and FSL-1 stimulation (Figure 1),demonstrating the high efficacy and selectivity of this inhibitor also for these experimental conditions. With live bacteria challenge, CU-CPT9a strongly attenuated the cytokine induction by GBS and *S. aureus*, for both time points and bacterial doses examined (Figure 1, Figure S2). Production of IL-1β and IL-12p70 induced by these bacteria was almost eliminated (Table 1A). TLR8 inhibition also strongly reduced TNF and IL-6 induction by GBS and *S. aureus*, and reduced IL-10 production at the late time point, while TLR8-inhibition increased the level of IL-8 after 22 h of infection with the highest dose of bacteria (Table 1A). In contrast, cytokine induction by ECO 1946 was not much affected by TLR8 inhibition. This is in accordance with previous findings using this particular strain, where TLR8 silencing failed to attenuate cytokine production in monocyte-derived macrophages (MDMs) (10). CU-CPT9a reduced the induction of TNF, IL-6, and IL-10 by the SAU 113-Δ*lgt* strain slightly more than with the SAU 113 wild-type strain (Table 1A). This suggest that TLR2 only plays a minor role in the sensing of *S. aureus* by monocytes compared to TLR8 for these experimental settings.

**Table 1:** Percentage reduction in cytokine release by TLR8 inhibition during bacterial challenge of monocytes.

(**A**) Infection with high/low dose of bacteria strains for 6- and 22 h (***N*** = 4). The doses were 5 × 10^**6**^/1 × 10^**6**^ per ml for GBS and ***S. aureus***, and 1 × 10^**6**^/2 × 10^**5**^ per ml for ***E. coli***.						
**Strain (6 h)**	**IL-12p70**	**IL-1β**	**IL-6**	**TNF**	**IL-10**	**IL-8**
GBS NEM316	**100**/**100**	**98**/**99**	**86**/**96**	**78**/**88**	5/**48**	−5/**48**
SAU 113-wt	**100**/**100**	**96**/**97**	**60**/**83**	**48**/**69**	−81/−42	−40/33
SAU 113-*dlgt*	**100**/**100**	**99**/**99**	**91**/**95**	**78**/**86**	−80/−11	−35/**43**
ECO 1946	54/28	29/27	−17/25	−10/20	−30/−27	−56/2
(22 h) GBS NEM316	**100**/**99**	**98**/**99**	**85**/**98**	**75**/**91**	**96**/**98**	–**200/**47
SAU 113-wt	**100**/**100**	**95**/**98**	**61**/**89**	53/**78**	59/**89**	–**215**/23
SAU 113-*dlgt*	**100**/**100**	**99**/**99**	**90**/**98**	**81**/**91**	**97**/**98**	–**242**/46
ECO 1946	77/49	37/35	−14/29	−5/27	−11/33	–**120**/18
(**B**) Infection with high/low dose of clinical bacteria isolates for 18 h (*N* = 5). The doses were 5 × 10^6^/1 × 10^6^ per ml for the Gram positive bacteria, and 1 × 10^6^/2 × 10^5^ per ml for the Gram negative species.						
**Isolate (18 h)**	**IL-12p70**	**IL-1β**	**IL-6**	**TNF**	**IL-10**	**IL-8**
GBS 248	**99**/**99**	**98**/**99**	**90**/**96**	**90**/**97**	**93**/**97**	−51/56
GBS 250	**99**/**99**	**99**/**98**	**85**/**91**	**89**/**97**	**92**/**92**	–**134**/36
SAU 17-2	**95**/**100**	**93**/**98**	42/**89**	62/**93**	**81**/**92**	–**324**/−6
SAU 17-3	**93**/**98**	**95**/**97**	48/**86**	65/**89**	**82**/**87**	–**289**/−19
SPN 18-1	**92**/**97**	**70**/**78**	44/**66**	72/**87**	46/**64**	−44/**32**
SPN 38	**98**/**99**	**93**/**91**	67/**69**	**89**/**89**	**75**/**72**	−56/35
ECO 17-1	55/32	35/23	−1/20	−2/46	27/−26	−44/66
ECO 18-1	**81**/58	58/36	−19/24	12/32	−20/−21	−55/32
PSA 17-1	**92**/**93**	**81**/**78**	−5/29	52/60	32/28	–**147**/28
PSA 17-2	**86**/**88**	72/63	−10/20	54/50	39/14	−126/29
(**C**) Infection with *E. coli* isolates (1 × 10^7^-1 × 10^6^-1 × 10^5^ per ml) for 5 h (*N* = 8–10).						
**Isolate (5 h)**	**IL-12p70**	**IL-1β**	**IL-6**	**TNF**	**IL-8**	
ECO 17-1	81/75/37	**48**/**37**/**34**	**39**/**47**/**42**	**44**/**40**/**34**	**20/31/25**	
ECO 18-1	**87**/**89**/62	**62**/**56**/**42**	**50/41**/**48**	**46**/31/21	1/-7/-1	

**Figure 2:**
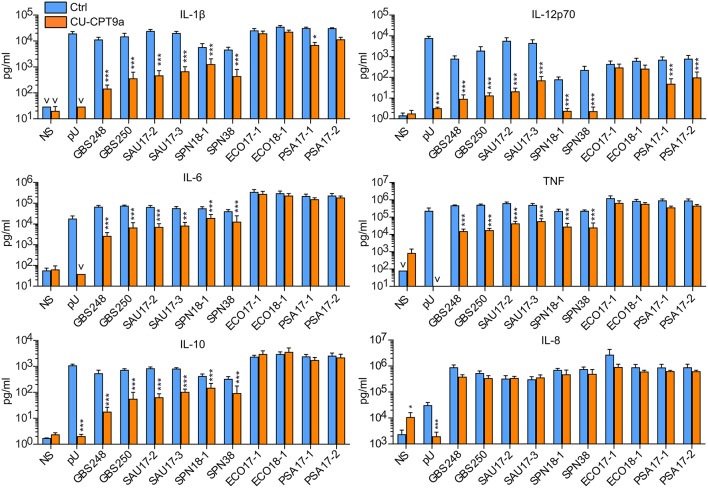
TLR8 inhibition strongly attenuates cytokine production from human primary monocytes challenged with clinical isolates of Gram-positive bacteria, while for the Gram-negative species *P. aeruginosa* (PSA) and *E. coli* this has less effects. Monocytes were pre-treated with control reagent (Ctrl, 5 μM) or TLR8 antagonist (CU-CPT9a, 5 μM) and challenged with TLR8-ligand pU or two clinical isolates of GBS, SAU, *S. pneumonia* (SPN), *E. coli* (ECO), and PSA for 18 h. The dose of Gram-positive bacteria was 1 × 10^6^/ml (MOI 0.1 for GBS, 0.2 for SAU, and 0.5 for SPN). The dose of Gram-negative bacteria was 2 × 10^5^/ml (MOI 0.2). Graphs show mean + SEM (*N* = 5). NS, no stimuli. *p < 0.05, **p < 0.01, and ***p < 0.001.

*The values correspond to the data in Figures 1, 2, and Figures S2, S3, and the effects that are statistical significant (p < 0.05) are shown in bold*.

### The Role of TLR8 During Challenge of Monocytes With Clinical Bacteria Isolates

We further examined the role of TLR8 in bacterial sensing using clinical isolates of GBS, *S. aureus, S. pneumonia, E. coli*, and *P. aeruginosa*. Cytokine production was determined 18 h after initiating the challenge, with two doses and two isolates per species. CU-CPT9a strongly reduced (70–100%) the production of IL-12p70 and IL-1β after challenging the monocytes with the Gram-positive species GBS, *S. aureus*, and *S. pneumonia* (Figure 2 and Table 1B). The inhibitor also clearly antagonized TNF, IL-6, and IL-10 production during Gram-positive infections, whereas the IL-8 levels increased by TLR8-inhibition for the highest bacterial dose. The limited effect of TLR8-inhibitor on the induction of IL-8 release by the bacteria reflects the relative weak induction of IL-8 by TLR8 agonist relative to TLR2 or TLR4 agonists (Figure 1, S2). In addition, IL-8 release is also efficiently released by cell stimulation with complement activation products (16). For the Gram-negative isolates, TLR8 blockade strongly reduced the IL-12p70 (86–93%) and IL-1β production (63–78%) during *P. aeruginosa* challenge. In comparison, CU-CPT9a reduced the cytokine levels less clearly upon challenge with clinical isolates of *E. coli*. Still, the production of IL-12p70 was reduced by up to 81% using CU-CPT9a and the highest dose of ECO 18-1 (Table 1B). We also examined the effects of CU-CPT9a during challenge with the *E. coli* isolates for 5 h. This revealed significant and non-redundant contribution of TLR8 to cytokine production during *E. coli* infection. Still, the percentage reduction in cytokine release by blocking TLR8 signaling is less for *E. coli* than for the other bacteria examined, and varies significantly among different strains and isolates of *E. coli*, as well as by the conditions examined (Table 1C and Figure S3). In conclusion, TLR8 appears as a dominant sensor of the Gram-positive isolates in monocytes, and it also plays a significant role for the detection of the Gram-negative isolates tested here.

### Cell Surface TLR Activation Limits TLR8-IRF5 Signaling and Induces a Rapid Loss of IRAK-1 Detection by Immunoblotting

**Figure 3:**
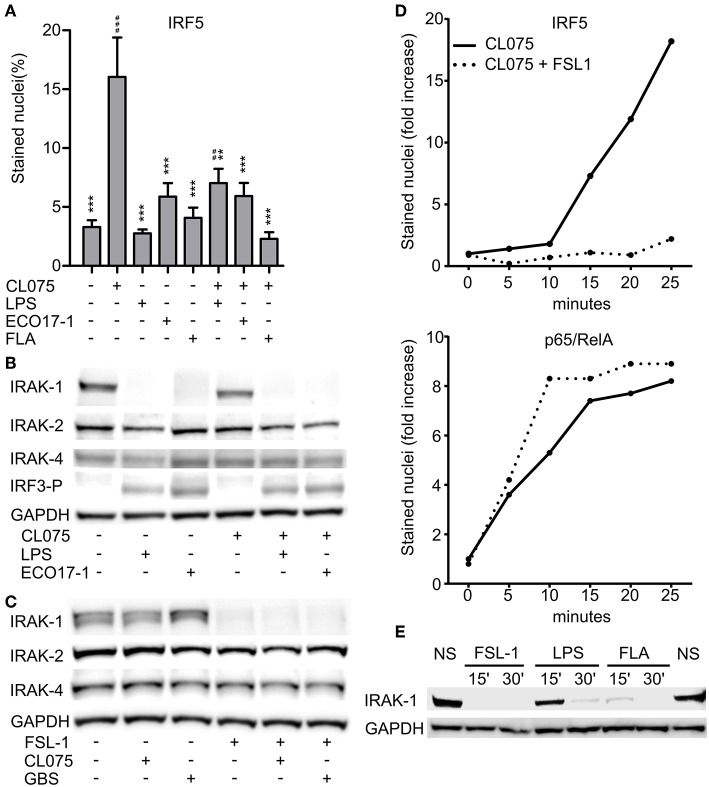
Surface TLR activation antagonize TLR8-IRF5 signaling and triggers a rapid loss of IRAK-1 detection on Western blot. **(A)** Levels of nuclear IRF5 in monocytes stimulated for 2 h with TLR8-agonist CL075 (1 μg/ml), TLR4-agonist LPS (1 μg/ml), ECO 17-1 (1 × 10^6^/ml), or TLR5-agonist Flagellin (FLA, 1 μg/ml) or combinations of these. Quantification of nuclear IRF5 was done by IF and scanR analysis (mean + SEM, *N* = 4).*indicate significant difference from CL075, while ^#^indicate significant difference from no stimuli. ^*##*^*p* < 0.01, ^*###*^*p* < 0.001, **p < 0.01, and ***p < 0.001. **(B)** Immunoblots of total IRAK-1, IRAK-2, IRAK-4, and IRF3-P after 30 min of treatment with CL075 (1 μg/ml), LPS (1 μg/ml), or ECO 17-1 (1 × 10^6^/ml) alone and in combinations. GAPDH served as a loading control, and a representative of four independent experiments is shown. **(C)** Immunoblots of total IRAK-1, IRAK-2, and IRAK-4 after 30 min of treatment with FLS-1 (100 ng/ml), CL075 (1 μg/ml), or live GBS (5 × 10^6^/ml) alone and in combinations. A representative of four independent experiments is shown. **(D)** Kinetics of IRF5 and p65/RelA nuclear accumulation in monocytes after treatment with CL075 (1 μg/ml) alone or combined with FLS-1 (100 ng/ml). Quantification of IRF5 and p65/RelA positively stained nuclei was done by IF and scanR analysis. A representative of four independent experiments is shown. **(E)** Effects of TLR stimulation for 15 and 30 min on IRAK-1 detection on immunoblot. Cells were untreated (NS) or stimulated with FSL-1 (100 ng/ml), LPS (1 μg/ml), and FLA (1 μg/ml). A representative experiment out of three independent experiments is shown.

We previously revealed that activation of TLR2 negatively regulates TLR8-TAK-1-IKKβ-IRF5 signaling in monocytes (9). Because *E. coli* is a weak activator of TLR8, we questioned if *E. coli* also can attenuate TLR8. To examine possible interference with TLR8-IRF5 signaling, we stimulated monocytes with CL075, and used *E. coli*, TLR4-, or TLR5- agonist as co-stimuli. CL075 activated TLR8-IRF5 signaling, but LPS and Flagellin did not. With ECO 17-1 infection there was a tendency for increased levels of nuclear IRF5 (Figure 3A), which might reflect a low TLR8-agonistic activity of this isolate. In co-stimulation with CL075, all three treatments (LPS, ECO 17-1, and Flagellin) significantly reduced the IRF5 nuclear accumulation (Figure 3A). Thus, similar to TLR2 activation (9, 10), signaling by TLR4 and TLR5 in human primary monocytes suppress TLR8-IRF5 signaling. Because *E. coli* expresses ligands for all three TLRs, this could impair TLR8-dependent recognition of this bacterium.

We next questioned if inhibition of TLR8 occurs at the level of proximal TLR signaling, at the Myddosome. Activation of IRF5 by TLR8 is dependent on the catalytic activity of IRAK-4 (17), and we were able to reproduced this finding using a specific IRAK-4 inhibitor (data now shown). We further examined the expression of total IRAK-1, IRAK-2, and IRAK-4 by immunoblotting after treatment with ECO 17-1, GBS or TLR ligands for 30 min. Challenge with ECO 17-1 or LPS triggered a loss of the IRAK-1 protein band that was detected in resting cells, while CL075 apparently had no effect, whether given alone or in combination with LPS or ECO 17-1 (Figure 3B). Still, quantification of IRAK-1 revealed a tendency for reduced IRAK-1 levels with CL075, while the levels of total IRAK-2 and IRAK-4 did not change during these conditions (Figure S4A). Both LPS and ECO 17-1 triggered phosphorylation of IRF3 at Ser396 (IRF3-P), thus correlating with the loss of the IRAK-1 band (Figure 3B). Stimulation with FSL-1 also induced loss of IRAK-1 detection, similar to LPS and ECO 17-1 (Figure 3C), even though FSL-1 does not induce IRF3 phosphorylation (10). In contrast to *E. coli*, GBS did not trigger the loss of IRAK-1 detection, and neither GBS nor CL075 influenced the TLR2-effect on IRAK-1. Again, the levels of IRAK-2 and IRAK-4 remained stable for all conditions (Figure 3C), while CL075 stimulation gave a tendency toward reduced IRAK-1 signal (Figure S4B). We next examined the early time kinetics of surface-TLR-mediated inhibition of TLR8-IRF5 signaling. IRF5 started to accumulate in the monocytes nuclei approximately 15 min after CL075 addition (Figure 3D). Co-stimulation with FSL-1 blocked the TLR8-IRF5 signaling already at this early stage. This indicates that TLR2 activation attenuates TLR8-signaling directly, and not via regulation of gene expression, translation, or autocrine/paracrine factors. In comparison to IRF5, CL075 stimulation increased the nuclear level of p65/RelA within 5 min, and co-stimulation with FSL-1 did not reduce but rather increased p65/RelA translocation (Figure 3D). Inhibition of TLR8-IRF5 signaling correlated with the rapid loss of IRAK-1 detection, which was observed 15 min after addition of FSL-1 and Flagellin, and 30 min after LPS (Figure 3E). We conclude that early signaling by surface TLRs differs from TLR8 at the level of IRAK-1, and the close correlation between the loss of IRAK-1 detection and the loss of TLR8-IRF5 activation indicates that the inhibitory crosstalk occurs at the Myddosome level.

### IRAK-1 Is Involved in Early TLR8-IRF5 Signaling, but Is Not Strongly and Rapidly Modified as in Surface TLR Signaling

**Figure 4:**
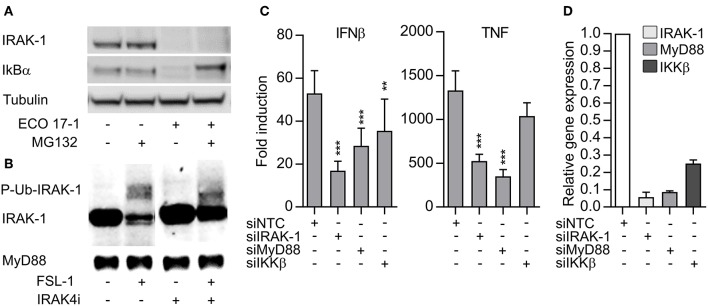
The loss IRAK-1 detection on immunoblot after *E. coli* infection is not caused by proteosomal degradation, and TLR8 signaling is dependent on IRAK-1. **(A)** Monocytes were treated with a proteasome inhibitor (MG132, 20 μM) for 30 min and then infected with ECO 17-1 (1 × 10e^6^ bacteria/ml) for 1 h. Total IRAK-1 and IkBα in cell lysates were examined by immunoblotting. Tubulin served as a loading control, and a representative of four independent experiments is shown. **(B)** THP-1 cells were differentiated, pre-incubated with IRAK-4 inhibitor (1 μM) or vehicle control, and stimulated with FSL-1 (100 ng/ml) for 30 min. Immunoblots of total IRAK-1 and MyD88 in lysates are shown in a representative of three independent experiments. **(C)** Human monocyte-derived macrophages (MDMs) were transfected with non-targeting control siRNA (NTC) or siRNA against IRAK1 (siIRAK-1), MyD88 (siMyD88), and IKKβ (siIKKβ). After 6 days, the cells were treated with TLR8 agonist CL075 (1 μg/ml) for 4 h. IFNβ and TNF expression were determined by quantitative PCR. *N* = 14. **p < 0.01 and ***p < 0.001. **(D)** Efficiency of gene silencing in MDMs. The experiment was performed as described above, without agonist treatment (*N* = 3). Graphs show mean + SEM.

TLR- and IL-1R-signaling can induce depletion of IRAK-1 via proteasomal degradation (18). On the other hand, detection of IRAK-1 on western blots may be lost because hyperphosphorylation and ubiquiylation of IRAK-1 during activation can slow down the migration of IRAK-1 and/or mask the antibody-binding epitopes (19). To clarify if IRAK-1 was degraded in our experimental model, we added the proteasome inhibitor MG132 and challenged the cells with *E. coli* for 60 min. The inhibitor efficiently blocked the degradation of IkBα, but the IRAK-1 band still disappeared (Figure 4A). This suggest that IRAK-1 detection is lost from the immunoblots after surface TLR activation due to covalent modifications such as phosphorylation and/or ubiquitination. Higher levels of IRAK-1 was found in lysates of THP-1 macrophages, and FSL-1 stimulation of these cells for 30 min induced changes in IRAK-1 migration, resulting in the detection of a number of larger IRAK-1 species on the western blots. Moreover, inhibition of the IRAK-4 catalytic activity partially reversed the changes in IRAK-1 migration (Figure 4B). These findings support the model of Vollmer et al. (19), where TLR/IL-1R-signaling results in rapid hyperphosphorylation and ubiquitlyation of IRAK-1, resulting in the loss of IRAK-1 detection by immunoblotting with some antibodies. The non-modified IRAK-1 band remained detectable 30 min after CL075 stimulation (Figures 3B,C), although there was a tendency toward a decrease (Figure S4). This questions whether IRAK-1 is involved or not in the early TLR8 signaling in primary myeloid cells. To clarify this, we silenced IRAK-1 in MDMs using siRNA and examined the effect on TLR8 signaling. Silencing of MyD88 and IKKβ were included as controls. Knockdown of IRAK-1 and MyD88 significantly reduced the induction of IFNβ and TNF after CL075 stimulation for 4 h (Figure 4C). IKKβ appeared less efficiently silenced (Figure 4D), but the partial silencing still reduced the induction of IFNβ by the TLR8-agonist, in agreement with an essential function of IKKβ in TLR8-IRF5 signaling (9).

### *E. coli*-Induced CD14/TLR4 Signaling Triggers IRAK-1 Modification and Limits TLR8-Dependent Sensing

**Figure 5:**
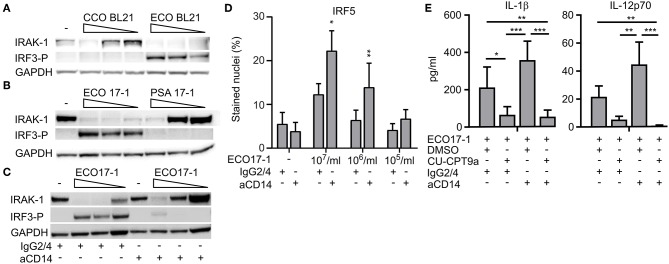
*E. coli* induced loss of IRAK-1 detection by immunoblotting is caused mainly by CD14- and TLR4-activation, and this limits TLR8-dependent sensing of *E. coli*. **(A)** The role of TLR4 in the loss of IRAK-1 detection during infection with *E. coli* was examined using the *E. coli* strain BL21 and its isogenic mutant Clear-Coli (CCO BL21), which does not activate TLR4. Monocytes were infected for 1 h with 1 × 10^7^-1 × 10^6^-1 × 10^5^ bacteria/ml, and the levels of total IRAK-1 and IRF3-P were determined by immunoblotting using GAPDH as loading control. A representative of three independent experiments is shown. **(B)** *E. coli* (ECO 17-1) induces loss of IRAK-1 detection more efficiently than *P. aerugionosa* (PSA 17-1), and correlates with less efficient TLR4-IRF3 activation and more efficient TLR8 activation by the latter species. Infection and immunoblotting were done as described above, and a representative of three independent experiments is shown. **(C)** Effect of a CD14 blocking antibody on *E. coli*-mediated loss of IRAK-1 detection. Monocytes were treated with anti-human CD14 (15 μg/ml) or its isotype control (IgG2/4) 30 min prior to infection. Infection with ECO17-1 and immunoblotting were done as described above, and a representative of three independent experiments is shown. **(D)** Effect of CD14-blocking antibody on ECO 17-1 mediated IRF5-activation. After 30 min of pre-treatment of monocytes with anti-CD14 and control IgG2/4, cells were infected with ECO 17-1 for 2 h. The level of nuclear IRF5 was quantified by IF and scanR imaging. Graphs show mean + SEM (*N* = 4). **(E)** Effect of combined CD14/TLR8 blocking on ECO 17-1-induced production of IL-1β and IL-12p70. Monocytes were pre-treated with anti-CD14, CU-CPT9a, appropriate controls, or combinations thereof for 2 h. Infection with ECO 17-1 (1 × 10^6^/ml) was done for 5 h, and the levels of IL-1β and IL-12p70 produced were determined with BioPlex. Graphs show mean + SEM (*N* = 6). *p < 0.05, **p < 0.01, and ***p < 0.001.

We next questioned whether *E. coli* evades TLR8 dependent sensing via surface-TLR-signaling that involves IRAK-1 modifications. To clarify the role of TLR4 in this process, we challenged monocytes with an LPS-deficient *E. coli* strain (Clear-Coli, CCO BL21) which does not activate TLR4. CCO BL21 did not activate IRF3 phosphorylation (Figure 5A), which confirms that it is an inefficient activator of TLR4. The mutant bacteria did not trigger loss of IRAK-1 detection at the lower doses (1 × 10^5^ and 10^6^/ml), while at the higher concentration (1 × 10^7^/ml) it did (Figure 5A). Hence, while TLR4 is central for the *E. coli*-induced modification of IRAK-1 at low concentrations of the bacteria, other sensors such as TLR2 and TLR5 might contribute, depending on the strain and conditions.

*P. aeruginosa* can also be sensed via TLR2, TLR4, and TLR5. Strains from the environment or adapted to laboratory, as well as clinical isolates from bloodstream or urinary tract infections, typically express penta-acylated LPS with low TLR4 agonist activity. In contrast, strains isolated from the airways of cystic fibrosis patients express hexa-acylated LPS that potently activates human TLR4 (20). For most *P. aeruginosa* strains, combined sensing via TLR4 and TLR2 might be important (21, 22). The *P. aeruginosa* isolates used here were from bloodstream- and wound- infections, suggesting that they express penta-acylated LPS. As they also seem to activate TLR8 more efficiently than *E. coli* (Figure 2), we questioned if this difference is due to less efficient activation of TLR4-MyD88-IRAK-1 signaling. Indeed, PSA 17-1 (blood culture isolate) did not induce phosphorylation of IRF3, and only triggered IRAK-1 modifications at the highest bacterial dose (Figure 5B). Therefore, the relatively strong activation of TLR8 by this bacterium correlates with less efficient signaling via TLR4-TRIF-IRF3 and TLR4-MyD88-IRAK-1.

We further examined the role of CD14 in *E. coli*-induced IRAK-1 modification, because CD14 is an important cofactor for both TLR2 and TLR4 (23). After 60 min of challenge with ECO 17-1, the CD14 blocking antibody attenuated the phosphorylation of IRF3 and increased the amount of non-modified IRAK-1 (Figure 5C), thus resembling the findings with PSA 17-1 (Figure 5B). Anti-CD14 further increased the activation of IRF5 during 2 h of challenge with ECO 17-1 (Figure 5D). This indicates that TLR8-dependent sensing of *E. coli* is suppressed by CD14/TLR4-MyD88-IRAK-1 signaling. Finally, anti-CD14 treatment enhanced the release of IL-1β and IL-12p70 during challenge with ECO 17-1 for 5 h, and the production of cytokines were largely TLR8-dependent in this condition, as demonstrated using the TLR8 inhibitor (Figure 5E). We conclude that sensing of *E. coli* via CD14/TLR4-IRAK-1 limits its sensing via TLR8.

## Discussion

We previously showed a role of TLR8 in the sensing of *S. aureus* (9) and GBS (10) in primary human monocytes and monocyte-derived macrophages, while TLR8 was not involved in the recognition of the *E. coli* strain Seattle 1946 (10). We here confirm and significantly extend these findings using a newly developed TLR8 antagonist with high selectivity and efficiency (14). We show that TLR8 is a dominant sensor of the Gram-positive species *S. aureus*, GBS, and *S. pneumonia* in human primary monocytes. TLR8 also contributes to non-redundant cytokine induction by clinical isolates of *P. aeruginosa* and *E. coli*, although it does not recognize the *E. coli* strain Seattle 1946.

While the impact of TLR8 in bacterial infections *in vivo* still is unclear, we find these new data interesting in the context of human MyD88- and IRAK-4 deficiency, where signaling by most TLRs and IL-1Rs is lost (24). During infancy and early childhood, but not in adulthood, these patients suffer from a strikingly narrow range of pyogenic infections caused by *S. aureus, P. aeruginosa*, and *S. pneumonia* (1, 2, 24). Hence, because TLR8 is central in the recognition of these bacteria by monocytes, the loss of TLR8 signaling may explain the increased susceptibility of these patients. Still, we do not know how important TLR8 is in the skin and the airway mucosa, the sites where these infections typically arise. Sensing via TLR2- and IL-1R-dependent mechanisms, may also be important here. Also, the loss of a single TLR is expected to result in incomplete penetrance of the clinical phenotype due to high redundancy and compensatory mechanisms (25). Besides TLRs, peptidoglycan fragments of both Gram positive and Gram negative bacteria can be sensed via the cytosolic sensors NOD1 and NOD2 (26, 27), and numerous studies have revealed that NOD- and MyD88-dependent signaling typically give synergistic responses (28). Moreover, the release of high levels of IL-1β by monocytes suggests that the bacteria may activate the NLPR3 inflammasome (29). In support of this, it was recently shown that the archaeon *Methanosphaera stadtmanae* is sensed via a TLR8-NLRP3 pathway in human myeloid cells (30). Taken together, we hypothesize that bacterial sensing via TLR8 and TLR2, in combination with IL-1R- and NOD/NLR-signaling, is critical in the defense against pyogenic infections in infants and children. The specific impact of TLR8 for the outcome of the complex host-pathogen interactions will likely vary, depending on genetic- and non-genetic factors of both the host and the pathogen.

We have earlier shown that TLR2 signaling inhibits TLR8-IRF5 activation, but the mechanism is not known (9). We here demonstrate that TLR2 co-stimulation blocks TLR8-IRF5 nuclear accumulation within 15 min. TLR4- and TLR5-agonists have a similar inhibitory effect, resulting in the attenuation of TLR8-IRF5-dependent cytokines release from monocytes, such as IL-12p70. In contrast, stimulation of human monocyte-derived DCs with LPS and R848 for 48 h gave synergistic IL-12p70 production (31). This may imply that the negative TLR4-TLR8-crosstalk in monocytes is overcome during the differentiation to DCs, while the mechanism behind is unknown.

Inhibition of TLR8-IRF5 signaling by surface TLRs correlates with their recruitment and activation of IRAK-1, as revealed by a rapid decline in the amounts of non-modified IRAK-1 with simultaneous appearance of hyperphosphorylated and ubiquitylated IRAK-1 species on western blot (19). In human cells, IRAK-4 is constitutively active and is autophosphorylated during the Myddosome assembly, while IRAK-1 is recruited by IRAK-4 and becomes activated upon dimerization (19). TLR8 does not trigger such rapid and extensive modifications of IRAK-1 as the cell surface TLRs, even though IRAK-1 is involved in TLR8-IRF5 signaling and IFNβ and TNF induction. We find that the IRAK-4 catalytic activity also is critical for TLR8-IRF5 signaling, in agreement with a previous study (17). Hence, IRAK-4-mediated phosphorylation of IRAK-1 may be essential in IRF5 activation. In contrast, inhibition of IRAK-4 catalytic activity only partially reduces NF-kB- and MAPK-signaling in human monocytes and murine macrophages, suggesting that these responses mainly require the structural function of IRAK-4 (17, 32).

The roles of IRAK-1 and IRAK-2 in MyD88-signaling are less clear than IRAK-4, and variable degrees of redundancy have been described in different cells and experimental conditions (19, 33–36). In murine dendritic cells, the catalytic activity of IRAK-1 was needed for TLR7- and TLR9-induced IFNα production (35, 36), while IRAK-2 mainly has a role in sustaining the response (35). In human MDMs and THP-1 cells, TLR-induced TNF release was dependent on IRAK-1 but not IRAK-2 (34). On the other hand, cells from an IRAK-1 deficient patient indicates that IRAK-1 and IRAK-2 are mainly redundant in TLR-induced pro-inflammatory cytokine induction in PBMCs, while IRAK-1 is non-redundant in TLR signaling in fibroblast (33). These results are partly conflicting with our data, which may be due to the different cellular contexts, experimental conditions and readouts. The IRAK-1 deficient PBMCs were stimulated for 36 h with R848 that also activates TLR7. Because pDCs and B-lymphocytes express TLR7 (37), activation of these cells within the PBMC population may have obscured the role of IRAK-1 in TLR8-signaling in monocytes. Also, we earlier showed that TLR7-induced IFNβ production in human monocytes is not affected by TLR2 co-stimulation (9), indicating that TLR7- and TLR8-signaling in monocytes differ. Our results suggest that IRAK-1 is particularly important in the early activation of TLR8-IRF5 signaling and IRF5-dependent cytokine induction, such as induction of IL-12p70 and IFNβ. We therefore propose a model of signaling where cell surface TLRs rapidly recruit and modify most of the IRAK-1 pool in the monocytes, and this may also include the sequestration of MyD88 and/or IRAK-4. This results in attenuation of TLR8-IRF5 signaling and IRF5-dependent cytokine induction (Figure S5). Similar to this model, TLR2-signaling suppressed TLR7-, and TLR9-induced IFNα production in murine BMDCs, which also correlated with loss of IRAK-1 detection on western blotting (38).

*E. coli* activates TLR8 less efficiently compared to the other bacterial species examined, including *P. aeruginosa*. CD14/TLR4-mediated detection of *E. coli* limits TLR8-IRF5 signaling according to the suggested model. In comparison, most *P. aeruginosa* strains activate TLR4 less efficiently, resulting in a more important role of TLR8 in detecting this bacterium. The Gram-positive species examined appear as even weaker activators of surface TLR signaling, and TLR8-dependent sensing is thus dominating. In addition to antagonizing TLR8-IRF5 signaling, cell surface TLRs trigger redundant cytokine production that also reduces the impact of TLR8-mediated bacterial sensing. Still, TLR8 contributed to increased cytokine production in response to the *E. coli* isolates, as most clearly seen for IL-12p70 and IL-1β. Production of these cytokines was also highly TLR8-dependent during infection with the other bacteria. In mice, IL-1β is critical for the resistance against experimental GBS and *S. aureus* infection, likely via IL-1R-mediated chemokine production which recruits neutrophils to the site of infection (39–41). TLR8-induced IL-12 production may be a critical signal for differentiation of follicular Th-cells and for efficient production of antibodies against invading bacteria such as *E. coli, Salmonella typhimurium*, and *Mycobacterium tuberculosis* (11). Even so, the functional impact of TLR8 in adaptive immunity in humans *in vivo* is still unclear, and patients with MyD88- or IRAK-4-deficiency appear not to be strongly impaired in antigen-specific T- and B-cell responses (1). Hence, further studies are required to clarify the different aspects of TLR8-mediated immunity and the functional consequences of the TLR-TLR negative crosstalk.

In conclusion, TLR8 is a major bacterial sensor in monocytes, and probably plays a more important role in the defense against bacteria than previously known. TLR8 seems dominant in the sensing of bacteria that avoid efficient activation of TLRs at the cell surface, such as staphylococci and streptococci. On the other hand, strong activation of cell surface TLRs by bacteria such as *E. coli*, limits TLR8 signaling, possibly via competition for Myddosome components. Nevertheless, TLR8 also contributes in the sensing of Gram-negative infections by monocytes.

## Data Availability

The datasets generated for this study are available on request to the corresponding author.

## Ethics Statement

Human buffycoats and serum were from the Blood bank at St. Olavs Hospital (Trondheim, Norway), with approval by the Regional Committee for Medical and Health Research Ethics (REC Central, Norway, no. 2009/2245).

## Author Contributions

SM, BE, JK, and JS planned and performed the experimental work, including cell isolation and cultivation, bacteria preparation and infection experiments. JS initiated the study, performed experiments with the TLR8-inhibitor, the scanR experiments, produced the final figures, and wrote the manuscript with help from SM and BE. SM did the silencing experiments and the immunoblotting. BE performed the experiments with the clinical isolates. JK did the bioplex analyses and contributed in the gene silencing experiments. MY assisted in the work on the signaling mechanisms and the discussion. KB contributed with discussion and interpretation of the work. JA provided the clinical isolates with recommendations for bacterial growth. ZH and HY provided the TLR8-inhibitor and control reagent and recommendations for their use. JD and TE contributed to the conception and interpretation of the work. All authors have read and approved the final version of the manuscript.

### Conflict of Interest Statement

The authors declare that the research was conducted in the absence of any commercial or financial relationships that could be construed as a potential conflict of interest.

## Supplementary Material

Figure S1 Efficiency of the TLR8 inhibitor CU-CPT9a in human primary monocytes. **(A)** Monocytes were pre-treated for 2 h with the control compound (Ctrl) or the TLR8 antagonist (CU-CPT9a) at different concentrations. Subsequently, the cells were stimulated with the TLR8-ligands pU/pLA (polyU/poly-L-arginine, 1 μg/ml) and CL075 (1 μg/ml). Supernatant was collected after 6.5 h and cytokine levels were determined with BioPlex. *N* = 2. **(B)** Chemical structure of CU-CPT9a and the control compound.

Figure S2 TLR8 inhibition strongly attenuates cytokine production from human primary monocytes challenged with Group B streptococcus (GBS) and *S. aureus*, but not a strain of *E. coli*. Cytokine levels were determined after 6 h of bacterial challenge. Bacterial doses were 5 × 10^6^/ml and 1 × 10^6^/ml for GBS and *S. aureus* (MOI 0.5/0.1 for GBS, 1.0/0.2 for SAU), and 1 × 10^6^/ml and 2 × 10^5^/ml for *E. coli* (MOI 1.0/0.2). Graphs show mean + SEM (*N* = 4). NS, no stimuli. ^*^*p* < 0.05, ^**^*p* < 0.01, and ^***^*p* < 0.001. For additional experimental details, see the Figure 1 legend.

Figure S3 TLR8 inhibition significantly reduces cytokine release from human primary monocytes challenged with *E. coli* isolates for 5 h. Cytokine release by monocytes pre-treated with control reagent (DMSO) or TLR8 antagonist (CU-CPT9a, 5 μM) and challenged with three doses of *E. coli* (ECO 17-1 and ECO 18-1): 1 × 10^7^/ml, 1 × 10^6^/ml, and 1 × 10^5^/ml (MOI 10.0, 1.0, and 0.10). Graphs show mean + SEM (*N* = 8–10). NS, no stimuli. ^*^*p* < 0.05, ^**^*p* < 0.01, and ^***^*p* < 0.001.

Figure S4 Quantification of the levels of IRAK-1, IRAK-2, IRAK-4, and P-IRF3 on western blot analysis after activation of TLR2, TLR4, or TLR8, *E. coli*, and GBS for 30 min, as indicated. *N* = 4 **(A)** or *N* = 3 **(B)**, and the data correspond to Figures 3B,C. ^*^*p* < 0.05 and ^**^*p* < 0.01.

Figure S5 Model of TLR-mediated sensing of Gram negative and Gram positive bacteria and negative crosstalk of TLR-signaling. TLR8 is a major sensor of Gram positive bacteria, while cell surface TLRs, in particular TLR4, is most important for sensing of Gram negative species. TLR8-IKKβ-IRF5 signaling in monocytes depends on IRAK-4 kinase activity and IRAK-1. TLR8-IRF5 activation is important for production of IFNβ, IL-12p70, IL-1β, and TNF. Cell surface TLR signaling results in recruitment and modification of IRAK-1 within minutes, and it closely correlates with rapid attenuation of TLR8-IRF5 activation. A possible explanation for this negative crosstalk is the sequestration of the Myddosome-components by the cell surface TLRs.
