# Supplementary figures and images for: Human Toll-like Receptor 8 (TLR8) Is an Important Sensor of Pyogenic Bacteria, and Is Attenuated by Cell Surface TLR Signaling
Source: Front Immunol. 2019 May 31;10:1209. doi: 10.3389/fimmu.2019.01209 (PMC6554558; doi:10.3389/fimmu.2019.01209)

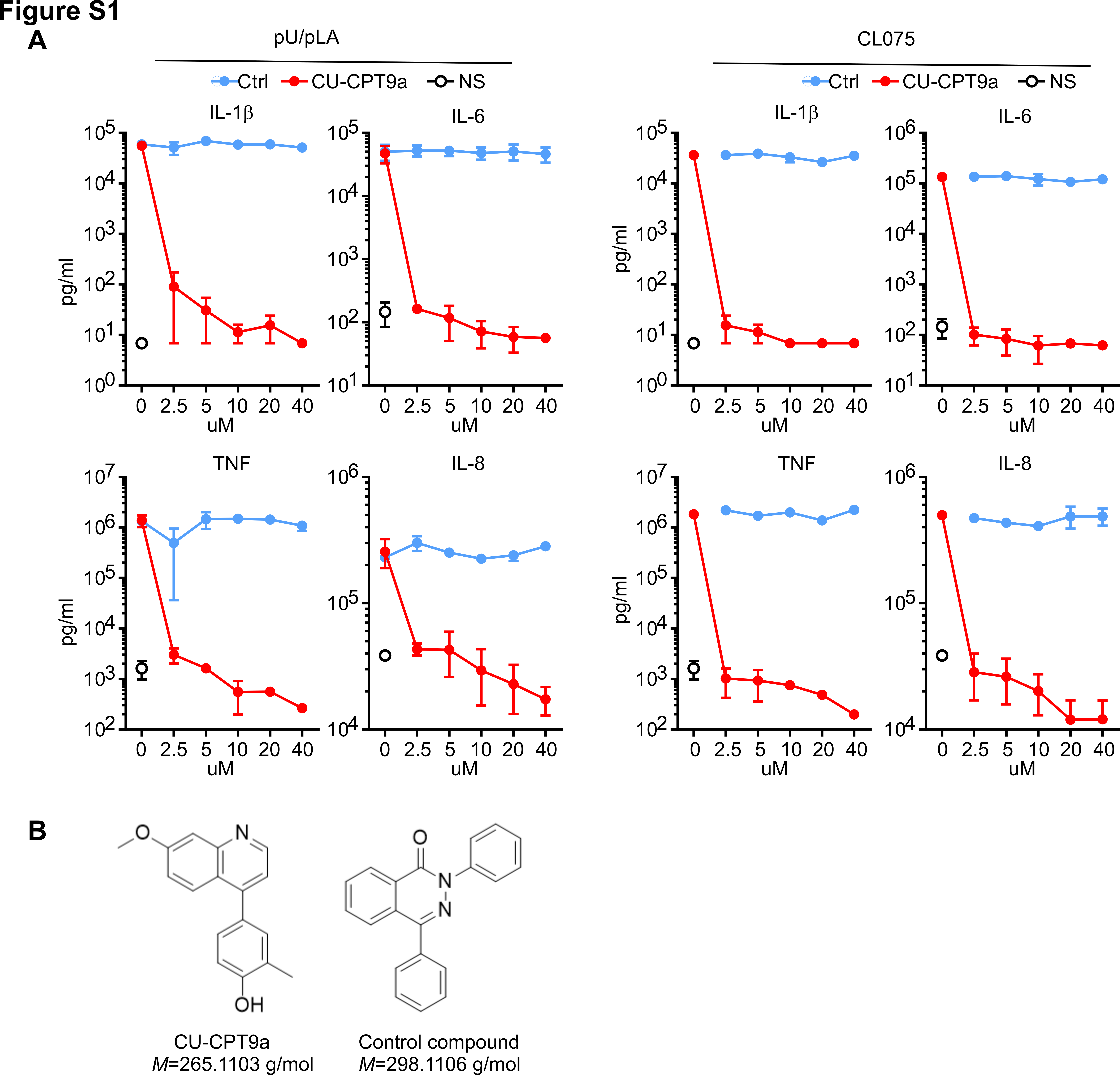

Supplement: Figure S1 — Efficiency of the TLR8 inhibitor CU-CPT9a in human primary monocytes. (A) Monocytes were pre-treated for 2 h with the control compound (Ctrl) or the TLR8 antagonist (CU-CPT9a) at different concentrations. Subsequently, the cells were stimulated with the TLR8-ligands pU/pLA (polyU/poly-L-arginine, 1 μg/ml) and CL075 (1 μg/ml). Supernatant was collected after 6.5 h and cytokine levels were determined with BioPlex. N = 2. (B) Chemical structure of CU-CPT9a and the control compound. [file Image_1.tif]

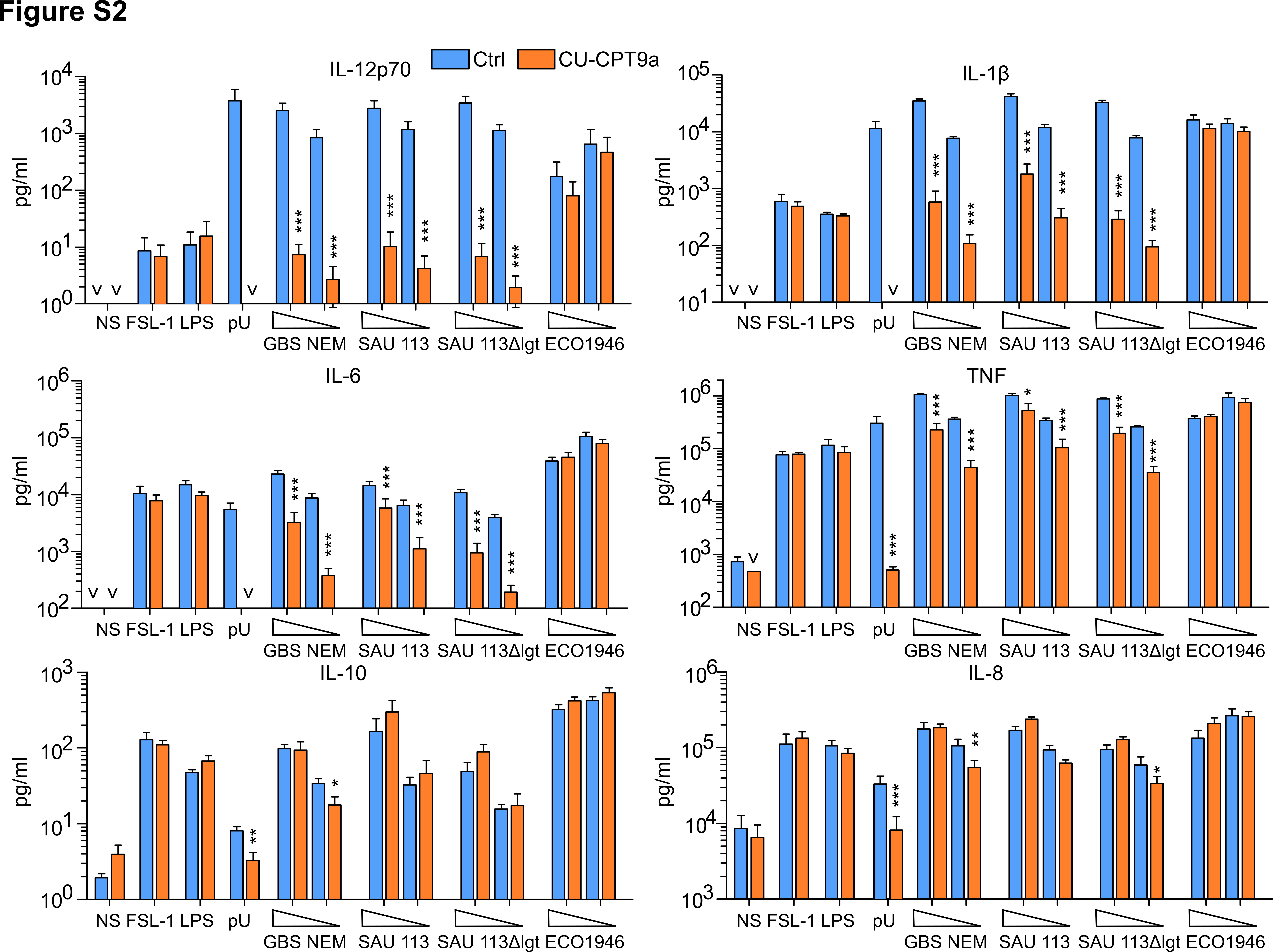

Supplement: Figure S2 — TLR8 inhibition strongly attenuates cytokine production from human primary monocytes challenged with Group B streptococcus (GBS) and S. aureus, but not a strain of E. coli. Cytokine levels were determined after 6 h of bacterial challenge. Bacterial doses were 5 × 10^6^/ml and 1 × 10^6^/ml for GBS and S. aureus (MOI 0.5/0.1 for GBS, 1.0/0.2 for SAU), and 1 × 10^6^/ml and 2 × 10^5^/ml for E. coli (MOI 1.0/0.2). Graphs show mean + SEM (N = 4). NS, no stimuli. *p < 0.05, **p < 0.01, and ***p < 0.001. For additional experimental details, see the Figure 1 legend. [file Image_2.tif]

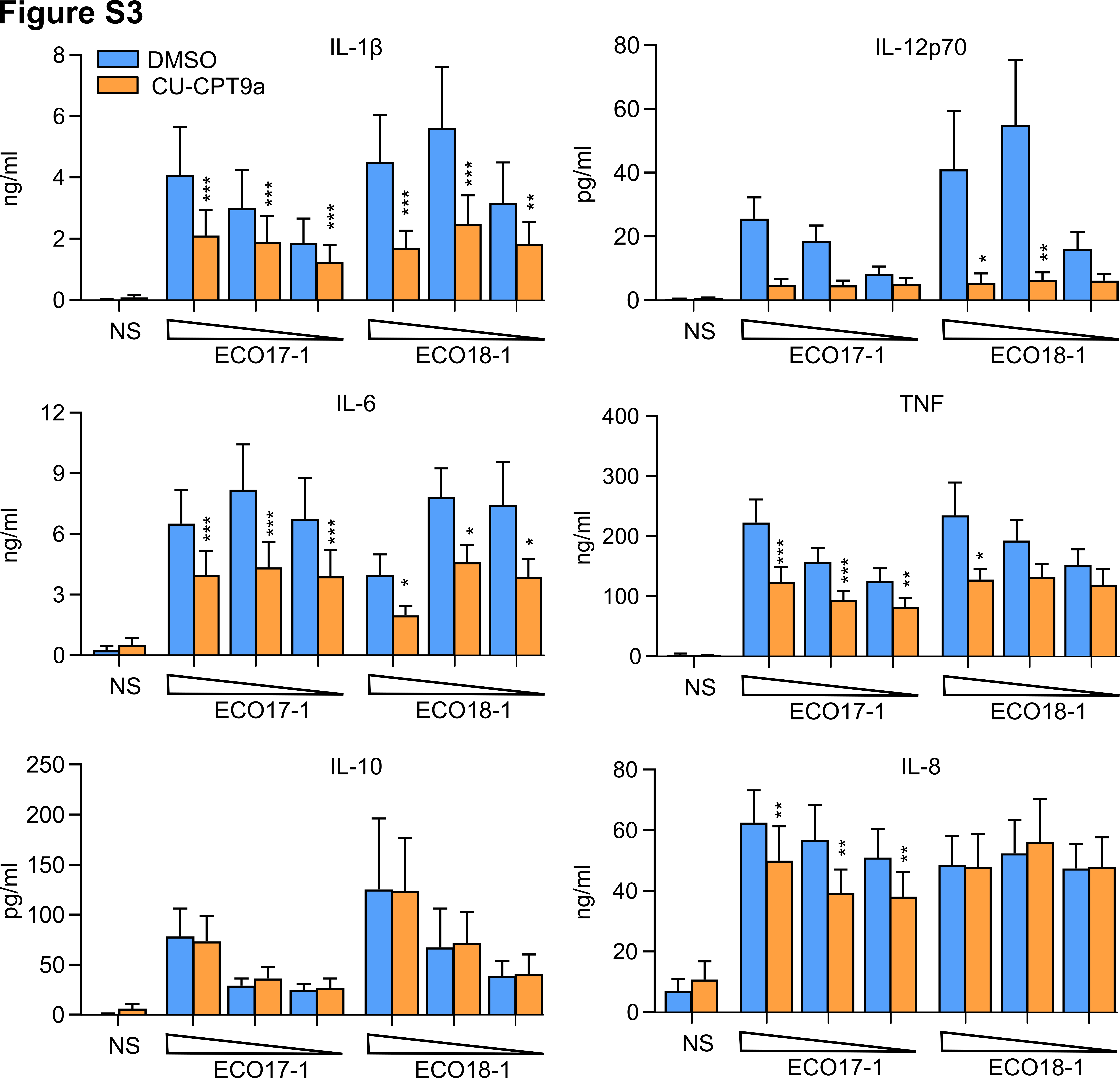

Supplement: Figure S3 — TLR8 inhibition significantly reduces cytokine release from human primary monocytes challenged with E. coli isolates for 5 h. Cytokine release by monocytes pre-treated with control reagent (DMSO) or TLR8 antagonist (CU-CPT9a, 5 μM) and challenged with three doses of E. coli (ECO 17-1 and ECO 18-1): 1 × 10^7^/ml, 1 × 10^6^/ml, and 1 × 10^5^/ml (MOI 10.0, 1.0, and 0.10). Graphs show mean + SEM (N = 8–10). NS, no stimuli. *p < 0.05, **p < 0.01, and ***p < 0.001. [file Image_3.tif]

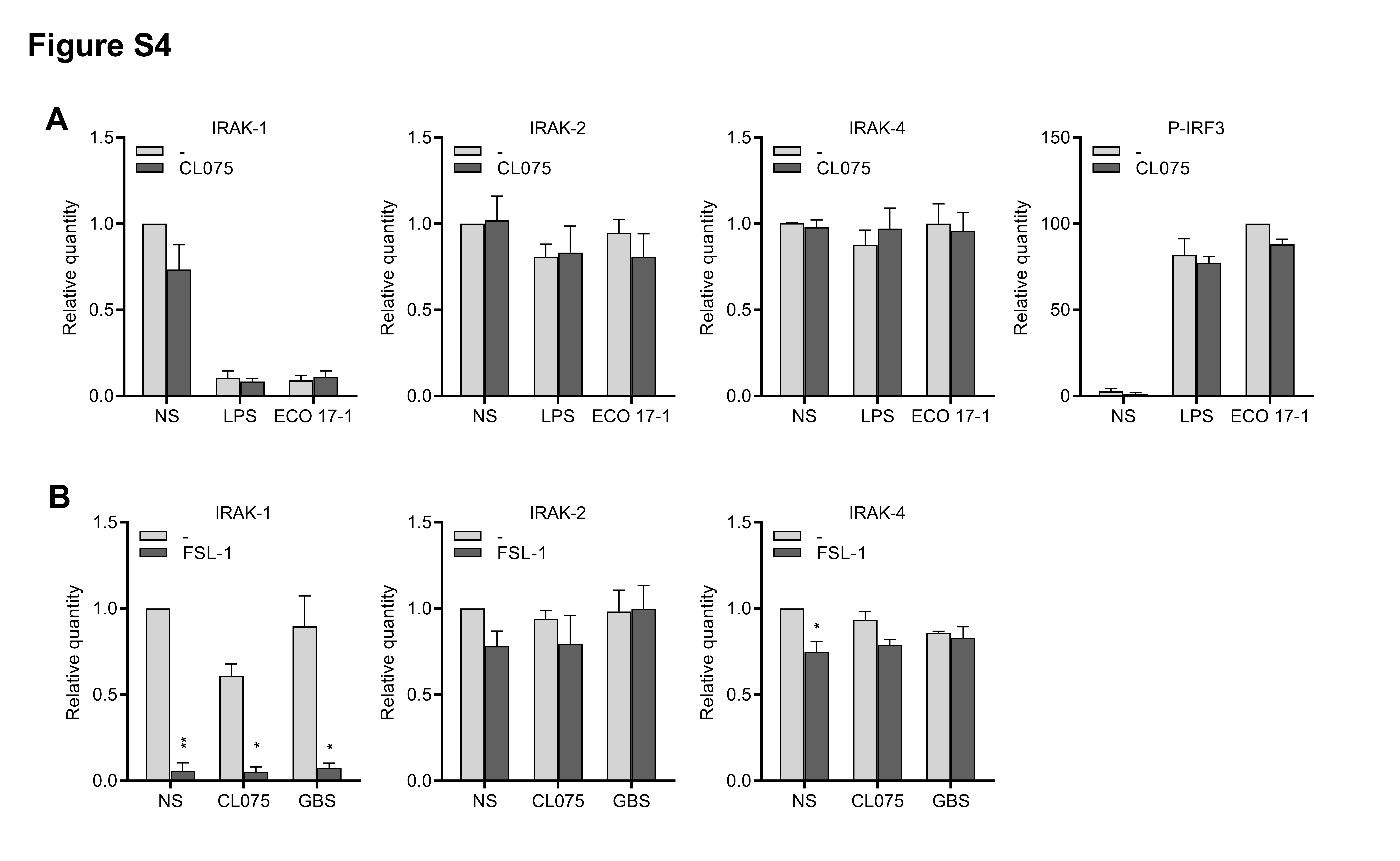

Supplement: Figure S4 — Quantification of the levels of IRAK-1, IRAK-2, IRAK-4, and P-IRF3 on western blot analysis after activation of TLR2, TLR4, or TLR8, E. coli, and GBS for 30 min, as indicated. N = 4 (A) or N = 3 (B), and the data correspond to Figures 3B,C. *p < 0.05 and **p < 0.01. [file Image_4.tif]

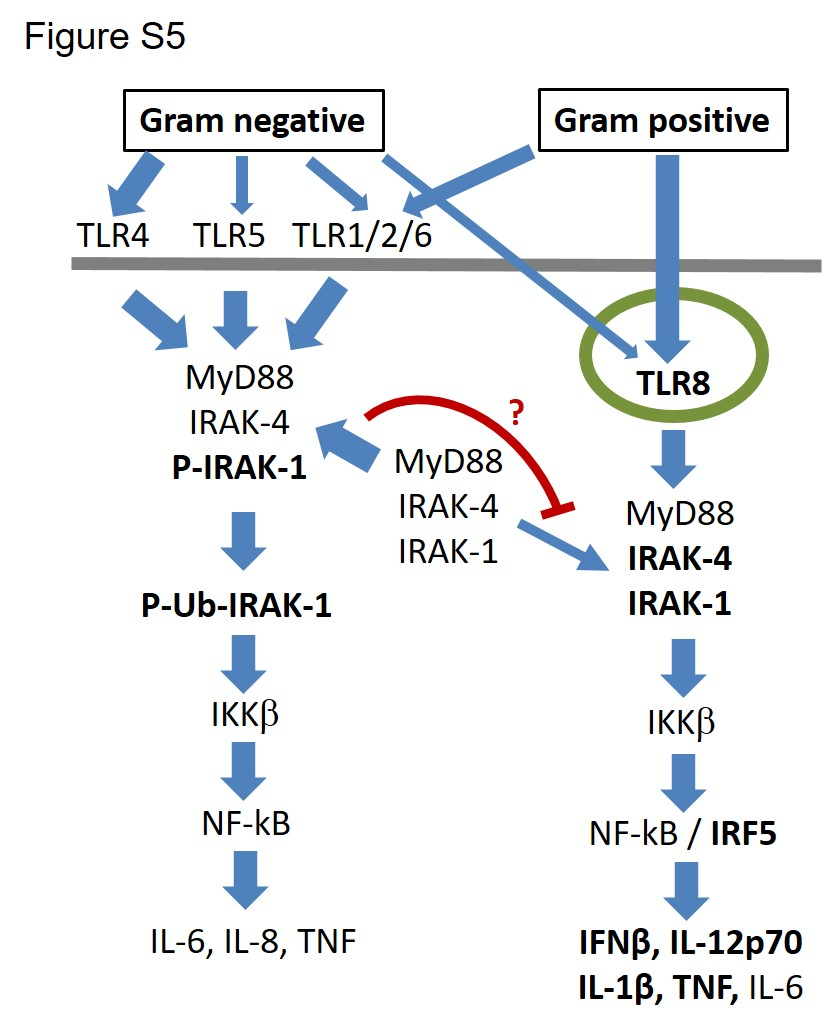

Supplement: Figure S5 — Model of TLR-mediated sensing of Gram negative and Gram positive bacteria and negative crosstalk of TLR-signaling. TLR8 is a major sensor of Gram positive bacteria, while cell surface TLRs, in particular TLR4, is most important for sensing of Gram negative species. TLR8-IKKβ-IRF5 signaling in monocytes depends on IRAK-4 kinase activity and IRAK-1. TLR8-IRF5 activation is important for production of IFNβ, IL-12p70, IL-1β, and TNF. Cell surface TLR signaling results in recruitment and modification of IRAK-1 within minutes, and it closely correlates with rapid attenuation of TLR8-IRF5 activation. A possible explanation for this negative crosstalk is the sequestration of the Myddosome-components by the cell surface TLRs. [file Image_5.tif]
